# Mechanisms of Action of Non-Steroidal Anti-Inflammatory Drugs (NSAIDs) and Mesalazine in the Chemoprevention of Colorectal Cancer

**DOI:** 10.3390/ijms140917972

**Published:** 2013-09-03

**Authors:** Carmine Stolfi, Veronica De Simone, Francesco Pallone, Giovanni Monteleone

**Affiliations:** Department of Systems Medicine, University of Tor Vergata, Via Montpellier 1, Rome 00133, Italy; E-Mails: ve.desimone@gmail.com (V.D.S.); pallone@uniroma2.it (F.P.)

**Keywords:** aspirin, non-steroidal anti-inflammatory drugs, 5-aminosalicylic acid, cyclooxygenase, prostaglandins, colitis-associated colon cancer, carcinogenesis, Wnt/β-catenin, cell cycle, DNA replication fidelity

## Abstract

Colorectal cancer (CRC) is the third most common malignant neoplasm worldwide. Although conclusive evidence is still lacking, epidemiologic studies suggest that long-term use of non-steroidal anti-inflammatory drugs (NSAIDs) has chemopreventive properties against CRC. Similarly, regular consumption of mesalazine, a drug structurally related to NSAIDs, seems to reduce the risk of CRC in patients with ulcerative colitis. These observations are supported by a large body of experimental data showing the ability of such drugs to inhibit multiple pathways that sustain colon carcinogenesis. This review summarizes the current information on the molecular mechanisms by which NSAIDs and mesalazine could interfere with CRC cell growth and survival.

## 1. Introduction

**Figure 1:**
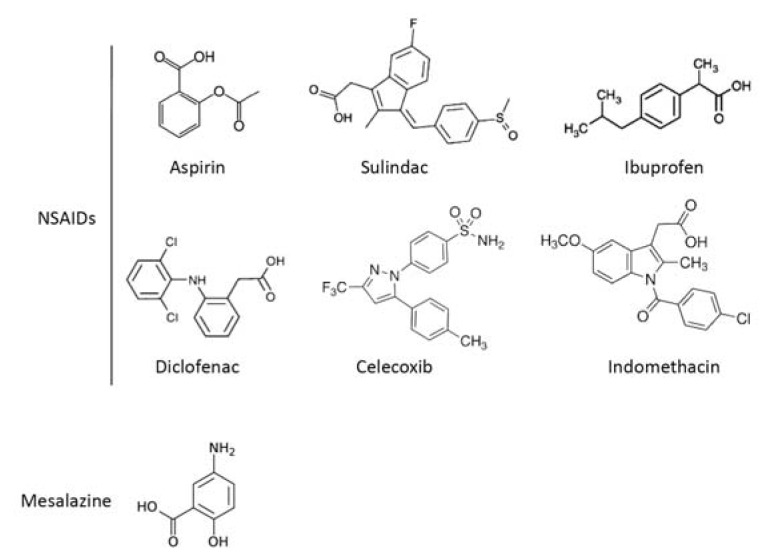
Chemical structures of common non-steroidal anti-inflammatory drugs (NSAIDs) and mesalazine.

Colorectal cancer (CRC) is one of the most common forms of malignancy and the second leading cause of cancer-related death in the Western world [1]. Sporadic CRC, occurring in individuals without any familial predisposition, represents the most common type of this neoplasia. The mechanisms that promote and sustain colon carcinogenesis are not yet known, though there is evidence that a complex interaction between environmental carcinogens and genetic alterations facilitates the selective growth of transformed cells, thus leading to the development of colonic dysplasia and cancer [2]. Colonoscopy screening, which is aimed at identifying and removing pre-cancerous lesions (*i.e.*, polyps), represents the gold standard of the preventive strategies for CRC, even though it is an invasive procedure that reduces the compliance and participation of CRC high-risk subjects in the screening programs. Another potentially complementary preventive strategy is based on the use of chemopreventive drugs. This option was suggested by large population-based studies showing that subjects who chronically take aspirin or other non-steroidal anti-inflammatory drugs (NSAIDs), such as sulindac, indomethacin and the selective COX-2 inhibitor, celecoxib, for various pathologies had a reduced risk of developing CRC [3–5]. Promising data on CRC chemoprevention have also recently emerged from epidemiological studies in patients with ulcerative colitis (UC), a clinical condition that is associated with enhanced CRC risk [6]. 5-Aminosalicylic acid (5-ASA), or mesalazine, a structural analogue of aspirin (Figure 1), is the drug of choice in the maintenance of remission in UC patients, and its long-term use was reported to reduce the incidence of UC-related CRC, though this protective effect was not confirmed in all of the studies [7–11].

In this article, we review the recent data on the molecular mechanisms underlying the protective effect of some commonly used NSAIDs and mesalazine in CRC.

## 2. Mechanisms of Action of NSAIDs in CRC Chemoprevention

Aspirin and NSAIDs have regulatory effects on the gene transcription and protein synthesis of multiple molecules involved in several inflammatory and neoplastic pathways [12]. In general, these effects can be differentiated based on the ability of aspirin and NSAIDs to suppress cyclooxygenase (COX) expression/activity and downstream signals, which are crucial for CRC cell growth, survival and diffusion. Therefore, the anti-neoplastic effects of NSAIDs and mesalazine will be discussed in the following paragraphs, taking into account both COX-dependent and COX-independent mechanisms.

### 2.1. COX-Dependent Mechanisms

COX enzymes (*i.e.*, COX-1 and COX-2) catalyze the conversion of arachidonic acid into prostaglandin (PG) G2, an unstable intermediate that is rapidly converted to PGH2. Subsequently, PGH2 is metabolized into different structurally-related PGs, including PGE2, PGD2, PGF2, PGI2 and thromboxane (TX)A2 [13]. COX-1 is constitutively expressed in mammalian tissues, and COX-1-derived PGs are essential for physiological functions, although studies in experimental models of CRC have suggested that COX-1 could exert tumor-promoting effects [14]. In contrast, COX-2 is inducible by inflammatory cytokines, growth factors and tumor promoters in several cell types [13]. Upregulation of COX-2 expression is seen in 40%–50% of human colorectal adenomas and in 80%–90% of carcinomas and results in enhanced PG production [15,16]. COX-2 plays a pivotal role in tumor initiation, promotion and progression by increasing the production of (1) reactive oxygen species, (2) PGE2 and other PGs that promote cell proliferation, (3) vascular endothelial growth factor and platelet-derived growth factor and (4) matrix metalloproteinases. COX-2 also controls the expression of both pro- and anti-apoptotic proteins and restrains the proliferation of immune cells with antineoplastic activity [17,18]. Among the NSAIDs, aspirin is the only drug that is able to permanently inhibit COX-1 and COX-2 activity. At anti-platelet therapeutic doses (75–100 mg daily), aspirin is up to 100-fold more potent in inhibiting platelet COX-1 than monocyte COX-2 [19]. Platelet activation is seen in CRC patients and is linked to key steps of colon tumorigenesis. Indeed, platelet activation increases the release of chemokines and proteolytic enzymes that support cancer cell proliferation, angiogenesis and metastasis [20]. Activated platelets might also contribute to COX-2 overexpression in CRC through the production of IL-1β, platelet-derived growth factor and TGF-β [20]. Therefore, the anti-tumorigenic effects of aspirin might rely, at least in part, on its anti-platelet action. The inhibition of COX-2 and PGE2 production by aspirin and other NSAIDs may rely on the modulation of various signals, including the inhibition of sphingosine-1-phosphate (S1-P) production and the stimulation of NSAID-induced gene (NAG-1). S1-P, a major product of sphingosine kinase-1, mediates angiogenesis, metastasis and protection of cancer cells from chemotherapy-induced apoptosis [21]. Nearly all human colon cancer cells express sphingosine-kinase-1, and S1-P stimulates COX-2 expression and PGE2-synthesis in CRC cells [22]. Aspirin (30–300 μM) and the non-selective COX-inhibitors, diclofenac (4–40 μM) and ibuprofen (200 μM), suppress S1-P release from human platelets *ex vivo* [23]. NAG-1 is a member of the TGF-β-superfamily that is involved in apoptosis and tumorigenesis [24]. NAG-1 expression is significantly reduced in both human and mouse CRC tissue compared to the normal intestinal mucosa [25]. Aspirin (1–10 mM) and other conventional NSAIDs (e.g., diclofenac (50–200 μM), indomethacin (50–100 μM) and the active sulfide form of sulindac, sulindac sulfide (5–50 μM)) upregulate NAG-1 in the CRC cell line, HCT-116 [26]. Using COX-2-deficient and NAG-1 transgenic *Adenomatous polyposis coli* (*Apc*)^Min/+^ mice, Iguchi and co-workers reported an inverse relationship between NAG-1 and COX-2/PGE2 in CRC and suggested that the induction of NAG-1 by celecoxib (1,500 ppm for four weeks) might contribute to the chemopreventive action of the compound [27].

### 2.2. COX-Independent Mechanisms

There is no doubt that the NSAID-mediated antitumor effects are not entirely dependent on COX inhibition. Indeed, not all COX-inhibiting NSAIDs possess anticancer properties, and restoring COX activity does not rescue CRC cells from NSAID-induced cell growth arrest. Moreover, NSAIDs can inhibit proliferation and induce apoptosis of COX-deficient CRC cells [28,29]. These observations have boosted intensive research aimed at elucidating which COX-independent signals can be regulated by NSAIDs in CRC cells. Such studies have shown that these compounds can, for example, regulate the activation of NF-κB. The NF-κB family includes various members that can associate to form homo- and hetero-dimers. Among these, the p50 and RelA (p65) heterodimer is bound in an inactive state in the cytoplasm by the inhibitor protein, I-kappaB (IκB) [30]. In response to activating stimuli, IκB is phosphorylated and then degraded by the proteasome, allowing the p50/RelA heterodimer to translocate to the nucleus, where it regulates the transcription of multiple genes [30]. Aspirin (5–10 mM) induces nucleolar sequestration of RelA in cultured CRC cells, thus resulting in decreased NF-κB transcriptional activity [31]. Consistently, aspirin (100–400 mg/kg) inactivates NF-κB *in vivo* in two mouse models of CRC, namely, the HT-29-derived xenograft model and the *Apc*^Min/+^ mice, and this effect is paralleled by the induction of apoptosis in tumor cells [32]. Sulindac sulfide (50 μM) inhibits HCT-116 cell invasion by suppressing the NF-κB-mediated transcription of specific microRNAs (e.g., miR-9, miR-17, miR-21) that modulate the expression of genes involved in tumor cell invasion and metastasis [33]. Inactivation of NF-κB in CRC cells can also be induced by indomethacin (125–250 μM) and sulindac sulfone (300–500 μM), a sulindac metabolite that lacks COX inhibitory activity [34].

NSAIDs can target the wingless and integration site growth factor (Wnt)/β-catenin pathway, which is constitutively activated in the majority of CRC cells. Wnt binds to the transmembrane Frizzled receptor, leading to activation of the cytoplasmic disheveled (Dsh) protein. Dsh associates with the β-catenin destruction complex, which consists of axin, Apc, protein phosphatase 2A (PP2A), glycogen synthase kinase 3 (GSK3) and casein kinase 1α (CK1α). In the absence of Wnt signaling, the destruction complex phosphorylates cytoplasmic β-catenin, thereby promoting its ubiquitination and degradation [35]. In contrast, in response to Wnt signals, degradation of β-catenin is decreased, and cytoplasmic β-catenin accumulates and is translocated to the nucleus, where it associates with T-cell factor (TCF) and lymphoid enhancer factor (LEF) family members to stimulate the expression of tumor-promoting genes (e.g., c-myc, c-jun, cyclin D1, matrilysin, peroxisome proliferator-activated receptor delta (PPARδ)) [35]. Aspirin (5 mM) and celecoxib (100 μM) increase the phosphorylation of β-catenin, thus decreasing its nuclear accumulation and the transcription of Wnt/β-catenin target genes in CRC cells [36,37]. Similar effects are reported for indomethacin (600 μM) and sulindac metabolites (*i.e.*, sulindac sulfide (120 μM) and sulindac sulfone (400 μM)) [38,39]. Additional evidence supporting the Wnt/β-catenin pathway as a target of NSAIDs in CRC chemoprevention comes from a recent report by Li and colleagues. This study showed that sulindac sulfide (50 μM) inhibits Wnt/β-catenin TCF transcriptional activity without increasing β-catenin phosphorylation, thus leading to the downregulation of cyclin D1 and the selective inhibition of CRC cell growth [40].

Acetylation is a phenomenon that allows for the post-translational regulation of protein functions [41]. In CRC cells, 100 μM aspirin acetylates the tumor suppressor protein, p53, thus increasing its DNA binding activity [42]. Additionally, aspirin (0.25–2.5 mM) acetylates and decreases the activity of glucose-6-phosphate dehydrogenase (G6PD), an enzyme involved in ribonucleotide biosynthesis and the positive control of tumor cell proliferation [43].

The DNA mismatch repair (MMR) system is a family of enzymes that recognizes and directly repairs nucleotide base mismatches, as well as other DNA damage-specific lesions, and the disturbed function of the DNA MMR system is associated with DNA microsatellite instability (MSI) [44]. Aspirin (2.5 mM) and sulindac (400 μM) reduce MSI in human CRC cells that lack DNA MMR activity [45]. Aspirin (2 mM) also stabilizes DNA by preventing DNA strand breaks induced by oxidative stress [46]. Aspirin (1–10 mM) increases MMR protein expression and subsequent apoptosis in MMR-proficient CRC cell lines [47]. However, in a mouse model of CRC, oral aspirin administration (400 mg/kg) failed to reduce MSI and slightly increased life span [48]. To ensure the fidelity of cell division and to maintain genome stability, eukaryotic cells have evolved sensor mechanisms called cell cycle checkpoints, whose activation induces cells to arrest at specific stages of the cell cycle to repair DNA damage and/or cell cycle defects [49]. Aspirin (2.5–10 mM) activates the cell cycle checkpoint-related protein, ataxia-telangiectasia mutated kinase (ATM), in CRC cells. This effect results in the upregulation of the cell tumor suppressor proteins, p53 and p21^Waf1/Cip1^, and cell arrest in the G1 phase of the cell cycle [50].

**Figure 2:**
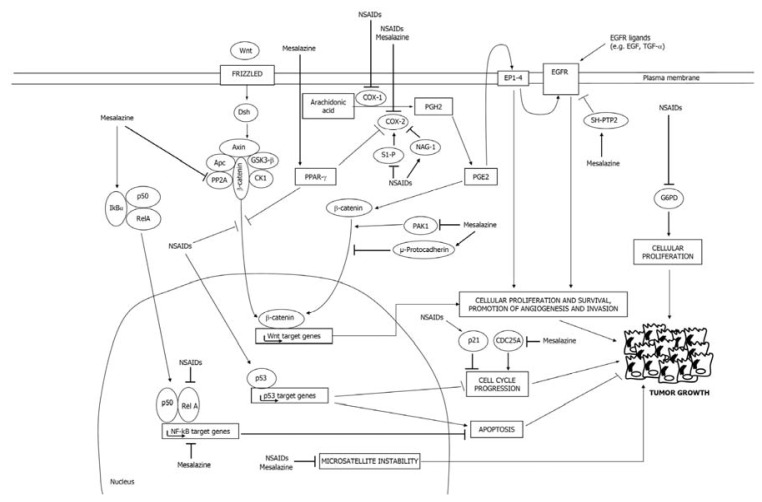
The figure illustrates some of the major molecular pathways regulated by NSAIDs and mesalazine in colorectal cancer (CRC) cells. Wnt, wingless and integration site growth factor; EGFR, epidermal growth factor receptor; PG, prostaglandin; S1-P, sphingosine-1-phosphate.

Some of the major molecular pathways regulated by NSAIDs in CRC cells are summarized in Figure 2.

## 3. Mechanisms of Action of Mesalazine in CRC Chemoprevention

Like NSAIDs, mesalazine can interfere with CRC cell growth and survival through COX-dependent and COX-independent mechanisms. Some of the major molecular pathways regulated by mesalazine in CRC cells are depicted in Figure 2.

### 3.1. Mesalazine Negatively Regulates the COX-2/PGE2 Axis

Mesalazine was initially shown to downregulate the COX-2/PGE2 axis in inflammatory cells that infiltrate the colonic mucosa of UC patients, which was assumed to be the basic mechanism by which the drug may prevent UC-associated CRC [51,52]. More recently, it has been demonstrated that mesalazine can also target CRC cells and inhibit pathways that are involved in sporadic CRC cell growth and survival. For example, mesalazine (15 mM) inhibits both basal and TNF-α/interleukin (IL)-1β-induced COX-2 expression in CRC cell lines, with the downstream effects of reducing PGE2 synthesis and cell growth [53]. However, the anti-proliferative effect of mesalazine is only partly reversed by exogenous PGE2 [53], raising the possibility that the anti-mitogenic effect of mesalazine may rely on the inhibition of additional pathways other than the COX-2 pathway. This hypothesis is supported by the demonstration that mesalazine (5–30 mM) inhibits the growth of DLD-1, a COX-2-deficient CRC cell line [53], and by studies in mice with colitis-associated CRC demonstrating that oral mesalazine (75 mg/kg) reduces the burden of both flat and polypoid dysplasia without affecting COX-2 expression [54].

### 3.2. Mesalazine Inhibits EGFR, NF-κB and Wnt/β-Catenin Signaling

Epidermal growth factor receptor (EGFR) is highly expressed by CRC cells, and its activation triggers mitogenic and pro-survival signals [55,56]. Mesalazine (25–50 mM) suppresses EGFR phosphorylation/activation in *ex vivo* organ cultures of human CRC explants and in CRC cell lines, and this inhibitory effect is not due to either shedding of the receptor or the inhibition of the synthesis of EGFR ligands [57]. In contrast, mesalazine enhances the activity of the protein tyrosine phosphatases (PTPs) that negatively control EGFR activation (*i.e.*, Src homology (SH)-PTP2) [57].

Mesalazine (50 mM) inhibits the TNF-α-stimulated degradation of IκBα and NF-κB activation in mouse colonic epithelial cells [58]. Additionally, mesalazine (5–40 mM) inhibits the NF-κB transcriptional activity induced in Caco-2 CRC cells by interleukin (IL)-1 or phorbol myristate acetate [59]. However, in Caco-2 cells, mesalazine does not prevent either IL-1-induced IκBα degradation or IL-1-induced nuclear translocation of NF-κB family members (e.g., RelA, RelB). Similarly, mesalazine does not interfere with the ability of NF-κB to bind to DNA sites. Overall, these observations indicate that mesalazine might inhibit NF-κB transactivation without interfering with the classic mechanism of regulation of this transcription factor. NF-κB transcriptional activity is also regulated by post-translational modifications (e.g., phosphorylation on tyrosine, threonine or serine residues) of NF-κB family members. Importantly, mesalazine seems to interfere with this alternative pathway of NF-κB regulation.

Like NSAIDs, mesalazine inhibits the Wnt/β-catenin pathway. In *Apc* mutated CRC cells with intact β-catenin, 1–2 mg/mL mesalazine (corresponding to 6.5–13 mM concentration) increases β-catenin phosphorylation and reduces the expression of Wnt/β-catenin target genes. The mechanism by which mesalazine enhances β-catenin phosphorylation is not fully understood, even though it was reported that mesalazine reduces the activity of the destruction complex-related protein, PP2A [60]. In CRC cells, mesalazine (20 mM) increases the expression of μ-protocadherin, a protein belonging to the cadherin superfamily that is able to sequester β-catenin on the plasmatic membrane, thus hampering its function [61].

The inhibitory effect of mesalazine on the Wnt/β-catenin pathway has been confirmed by *in vivo* observations. Mesalazine significantly reduces both β-catenin levels and its nuclear localization, as well as the expression of β-catenin target genes in sporadic adenomas [62]. Khare and colleagues showed that administration of 2,500 mg/kg mesalazine in mouse chow to *Apc*^Min/+^ mice (corresponding to three grams/day in humans) downregulates p21-activated kinase 1 (PAK1), a serine/threonine kinase required for the full activation of Wnt/β-catenin signaling [63].

### 3.3. Mesalazine Activates PPAR-γ in CRC Cells

The peroxisome proliferator-activated receptor gamma (PPAR-γ) is a transcription factor belonging to the nuclear hormone receptor superfamily. PPAR-γ is highly expressed in the colon and is known to regulate cellular proliferation, differentiation and apoptosis [64,65]. Activation of PPAR-γ inhibits the formation of aberrant crypt foci and the development of CRC in rodents [66,67]. Mesalazine (50 mM) enhances PPAR-γ expression in CRC cells and promotes its translocation from the cytoplasm to the nucleus [68]. Studies in immune-deficient mice engrafted with human CRC cells showed that locally administered mesalazine (50 mM) significantly reduced the growth of xenografts, and this effect was blocked by a selective PPAR-γ antagonist [69]. More recently, Schwab and colleagues confirmed that mesalazine (30–50 mM) enhances PPAR-γ expression and activity in HT-29 and Caco-2 cells [70]. Mesalazine-driven PPAR-γ upregulation was followed by the induction of the tumor suppressor gene, PTEN, activation of caspase-8 and caspase-3 and diminished expression of anti-apoptotic proteins (*i.e.*, survivin and the X-linked inhibitor of apoptosis protein) [70]. The anti-proliferative effect of mesalazine was partially, but not totally, reversed in the dominant-negative PPAR-γ HT-29 cells, thus confirming that further mechanisms besides PPAR-γ are involved in mesalazine-mediated CRC cell growth arrest and apoptosis.

### 3.4. Mesalazine Modulates Cell Cycle-Related Proteins

The inhibition of CRC cell growth by mesalazine is associated with the modulation of different replication checkpoints, thus resulting in the perturbation of cell cycle progression. In this context, we have shown that mesalazine (5–30 mM) causes a progressive accumulation of CRC cells in the S phase and decreases the percentage of cells in the G2/M and G0/G1 phases [71]. The S-phase cell cycle block of CRC cells following mesalazine treatment is preceded by the rapid ubiquitination and proteasome-dependent degradation of CDC25A, a phosphatase that is over-expressed in the majority of CRC cells and that is known to regulate the G1/S transition and S phase progression through the modulation of different cyclin/cyclin-dependent kinase (CDK) complexes [71]. These results are in line with those published by Luciani and colleagues, who showed that exposure of HCT-116 and HT-29 cells to mesalazine (5–40 mM) resulted in a reversible accumulation of cells in the S phase through a mechanism that is p53-independent and associated with the activation of proteins involved in the ATM-and-Rad3-related kinase (ATR)-dependent S-phase checkpoint response (e.g., Chk1, RAD17) [72]. However, other authors have shown that mesalazine can induce CRC cells to arrest in either the G0/G1 or G2/M phase of the cell cycle [73,74]. The reason for this apparent discrepancy is unknown, but it could reflect differences in the culture system adopted, including the dose and time of exposure to the drug.

### 3.5. Mesalazine Improves Replication Fidelity

Sporadic and UC-related CRC are characterized by very similar frequencies of the two major types of genomic instability, namely, chromosomal instability (CIN) and MSI [75]. CIN is characterized by the atypical segregation of chromosomes and abnormal DNA content (aneuploidy), which causes the loss of whole chromosomes or parts of chromosomes and, consequently, reduces the expression of critical tumor suppressor genes (e.g., *Apc*, *p53*). MSI is characterized by an increased rate of point mutations and is related to defects in the MMR system. During this process, frameshift mutations, called microsatellites, tend to accumulate. As microsatellites mainly occur in intronic DNA sequences, microsatellite mutations normally result in no gene function alterations. However, if microsatellites are located in exonic gene regions, they can lead to a shift in the codon reading frame, which ultimately results in a loss of protein function [76]. Frameshift mutations at microsatellites occur as a time-dependent function of polymerase errors, followed by the failure of post-replicational MMR. Studies performed by Gasche *et al.* demonstrated that mesalazine (5 mM) improves replication fidelity in cultured CRC cells [77]. In particular, these authors developed a flow cytometry-based assay that allows for the quantification of frameshift mutations at a (CA)13 microsatellite through the switch-off of a reporter protein. As the effect of mesalazine was seen in MMR-deficient CRC cells, it is likely that mesalazine acts on replication fidelity independently of the post-replicational MMR. Gasche’s group associated the molecular mechanism by which mesalazine inhibits the generation of frameshift mutations with the slowing down of DNA replication and cell division caused by this drug [72]. Indeed, cell cycle regulation is one of the defense mechanisms that allow cells to either repair DNA damage or eventually undergo apoptosis, thus safeguarding the integrity of the genome [78]. The same group also demonstrated that the ability of mesalazine to reduce replication errors in CRC cells was not limited to (CA)13 repetitive sequences, as the drug increased replication fidelity in other dinucleotide repeats, as well as in mononucleotide and tetranucleotide repeats [79].

## 4. Conclusions

Over the last few decades, a considerable amount of evidence has been accumulated to indicate that aspirin and other commonly used NSAIDs are effective in preventing CRC. The anti-neoplastic properties of these drugs are linked to their ability to inhibit the COX enzymes, as well as to modulate COX-independent pathways. However, the anticancer effects of NSAIDs often require higher concentrations (in the micromolar range) than those needed to inhibit COX (in the nanomolar range). While supporting the importance of the COX-independent mechanisms underlying NSAID anticancer activity, this discrepancy actually represents a major issue for the use of NSAIDs in CRC chemopreventive programs. Indeed, the long-term use of these drugs is associated with a relatively high risk of gastrointestinal and other side effects resulting from COX inhibition. In this context, it is worth noting that recent studies demonstrate the possibility of uncoupling the COX-inhibitory and anticancer activities of NSAIDs, thus supporting the development of new NSAID derivatives that could more safely achieve higher concentrations *in vivo*, following long-term administration [80].

Unlike NSAIDs, mesalazine-based therapy does not seem to be associated with serious adverse effects. Collectively, the available data show that this drug can directly modulate the activity of CRC cells. These observations, together with the demonstration that sporadic and colitis-associated CRC share major intracellular pathways that sustain the neoplastic process, suggest that mesalazine-based chemopreventive programs can be useful, not only in patients with longstanding and extensive UC, but also in individuals at high risk of developing sporadic CRC. Before clinical use, however, further studies will be necessary to optimize the dosage/concentration, route and delivery systems of the drug, as well as the treatment duration.
